# ”It´s like walking in a bubble”, nursing students´ perspectives on age suit simulation in a home environment – group interviews from reflection seminars

**DOI:** 10.1186/s12912-024-01792-5

**Published:** 2024-02-16

**Authors:** Björn Bouwmeester Stjernetun, Catharina Gillsjö, Elzana Odzakovic, Jenny Hallgren

**Affiliations:** 1 School of Health Sciences, University of Skövde, Box 408, SE-541 28 Skövde, Sweden; 2 School of Health and Welfare, Jönköping University, Jönköping, Sweden; 3 College of Nursing, University of Rhode Island, Kingston, RI USA

**Keywords:** Age suit simulation, Education, Nursing students, Ageism, Older persons, Welfare technology, Experiential learning theory

## Abstract

**Background:**

Older persons with age-related and complex health problems will increasingly depend on care provision from nurses in their own homes. However, a barrier to quality care is ageism and nursing students´ disinterest in geriatrics. In addition, nurse education often falls short in preparing students for the complexity of geriatric care. Welfare technology (WT) is progressively implemented in home care to help older persons live at home despite their health problems. However, this process is intricate and requires acceptance and digital literacy among caregivers and older persons. Despite these challenges, nurse education can address and change negative attitudes through innovative teaching methods such as age suit simulation. Therefore, the study aims to describe nursing students´ experiences of age suit simulation in a home-like environment with WT and technical aids, and will reveal their perspective on ageing and providing care to older adults.

**Methods:**

A qualitative explorative design using semi-structured group interviews (*n*=39) among nursing students. Data was analysed through reflexive thematic analysis.

**Results:**

The analysis generated three main themes; “It’s like walking in a bubble”, “An eye opener” and “Concerns about ageing and the current structure of geriatric care”. The main themes included eight subthemes. Adapting to the sensory and physical limitations of the age suit was an immersive experience and caused feelings of frustration, loneliness and disconnection. A prominent result was a raised awareness of cognitive loss, especially impaired vision, and students felt the simulations had made them aware of the everyday challenges older persons faced. Students highlighted the importance of patience and giving enough time in care situations by being present and having a critical perspective of WT. The students were mostly negative towards their own ageing and could better relate to older persons´ vulnerability.

**Conclusions:**

Age suit simulation was described as an embodied and eye-opening experience, raising nursing students´ awareness of older persons´ functional limitations and the consequences for dignity and independence. Coping with cognitive loss was especially difficult. Students were motivated to apply their new knowledge to clinical practice. Age suit simulation can complement geriatric education, preparing students for the complex care needs of older persons.

**Supplementary Information:**

The online version contains supplementary material available at 10.1186/s12912-024-01792-5.

## Background

The World Health Organization (WHO) acknowledges that the ageing global population presents challenges for future health care provision, where nurses are often the primary health care provider. Ageing is highly individualised but is primarily associated with various health problems, leading to difficulties in daily life and a need for health care interventions. Older persons today are generally healthier than their previous generations and wish to remain active members of society. However, ageist attitudes in society and the health care system negatively impact the quality of care older persons receive [1]. Ageism is a global issue and has consequences on both a structural and individual level, including denied access to health care services, exclusion from research, shorter life expectancy, less likelihood of recovering from injury, mental illness and declining social life and relationships [2].

Since the early 2000s, there has been a shift from residential care for older persons in favour of home care, signalling that older persons should receive care in their own homes [3]. This trend, coupled with the use of digital solutions, necessitates innovations in home care provision [4, 5] and the need for nurses to be comfortable with any technology associated with patient care [6]. Welfare technology (WT) is often implemented with the intention of helping older persons to live at home despite complex health care problems. There is no universal definition of WT, but it generally refers to digital technology used in health and social care with the aim of preserving and increasing safety, activity, participation or independence for persons with function disabilities [7]. Implementing WT is intricate and requires acceptance and digital literacy among caregivers and older persons [8]. Despite the widespread use of WT in healthcare, nursing students express the need for increased understanding of this technology through practical training and interaction during their education [9]. Nurse educators should therefore incorporate content in the curriculum to enhance students´ digital literacy [10].

In geriatric care, nurses must understand the older persons' perspective and, at times, advocate for their patients. Crucial skills in this context include effective communication, thoughtful care planning, and a solid knowledge base in gerontology and geriatrics, enabling nurses to monitor and assess a patient’s health and cognitive status [11]. But a challenge to the health care needs of an ageing population is a global nurse shortage, where it is estimated that the number of nurse graduates must increase by 8% in order to meet global health care demands by 2030 [12].

In addition, geriatrics is often rated unfavourably as a career choice among nursing students. Reasons for this perception include the complexity of care and the belief that the work can be slow-paced and depressing [13]. Nursing students with low interest in geriatric care tend to have less favourable attitudes towards older persons [14] and these attitudes can be part of a distancing culture, where young persons avoid thoughts of ageing and perceive little meaning in growing old [15]. Fear of ageing and death are also related to reduced optimism among young persons [16]. Therefore, nursing students need motivation and guidance to see that caring for older persons is complex, stimulating, and skill-intensive [17]. Challenging experiences can help students develop ethical awareness and identify ethical issues in practice [18]. However, students are often ill-prepared for geriatric care as courses often focus on skills rather than the complexity of care and healthy ageing, especially when it comes to caring for persons with cognitive impairments [19].

Despite these challenges, nursing education can change students´ attitudes. Simulations involving experiential learning are particularly effective in empathy training [20]. The use of age suit simulations where students experience the perspective of an older person dealing with health issues is one such intervention that has proven to be effective in increasing nursing students´ empathy [21] and positive attitudes towards older persons [22]. An age suit is composed of components that simulate age-related impairments and health problems that affect older persons, for example reduced mobility, muscle mass and grip strength, arthritis, chronic obstructive pulmonary disease, hearing loss and impaired vision. Younger persons who wear the age suit can therefore experience the age-related impairments and health problems of older persons [23]. To our best knowledge no study has investigated age suit simulation in the context of home like environment where students also interact with WT. The context of home and WT in combination with age suit simulation is highly relevant in nurse education, especially in light of the ongoing change internationally [24] and nationally [25] with the transfer of provision of care from institution to home. This ongoing transition will change the working environment for future nurses. Therefore, this study aims to describe nursing students´ experiences of age suit simulation in a home-like environment with WT and to reveal their perspective on ageing and providing care to older adults.

## Method

### Research design

A qualitative explorative design was chosen using semi-structured group interviews among nursing students. According to Polit and Beck [26] semi-structured group interviews are useful when the researcher wants to generate a comprehensive dialogue about specific topics. The interview guide that was developed for this study is provided as a supplementary file (Appendix 1).

### Sampling and recruitment

The recruitment of participants began during the period 2019–2022 when nursing students during their second academic year at a university in the western region of Sweden participated in an age suit simulation at Skaraborg Health Technology Center (SHC). The participants were recruited by convenience sampling. The inclusion criteria for participation were: to be nursing students, enrolled in the nursing program, during their second academic year. Fourteen to 16 students, divided into two groups, participated in four-hour sessions, either in the morning or afternoon, involving age suit simulation followed by group interviews. The group interviews were conducted in both the morning and afternoon sessions.

### Simulating in the age suit at SHC

The GERonTologic age suit (GERT) [23] was used by the nursing students to simulate ageing and age-related health problems. The GERT suit comprises different accessories such as straps, weights, eyeglasses, gloves and headphones that simulate age-related and specific health problems, for example hemiparesis, impaired vision and eye diseases, tremor, tinnitus and overall restricted movements and unstable gait. In the present study, the students were assigned one out of eight *personas* (a character with specific health problems created from the age suit). During the simulations at SHC the students were instructed to perform ordinary tasks in an apartment at SHC, such as getting into and out of bed, brushing their teeth and combing their hair, writing a simple message, cleaning and setting the table. During the task, the students interacted with the apartment’s WT and aids. The simulations lasted for approximately 50 minutes.

### Data collection

Data collection through group interviews was a collaborative effort carried out by B.B.S., C.G. and J.H. In total, 59 group interviews were conducted and recorded during the period 2019 to 2022, of which 39 interviews were selected for inclusion in this study. The selection process involved systematically choosing one interview from the morning class and one interview from the evening class from each scheduled simulation. To ensure a rich and representative dataset, the selected group interviews cover a timeframe that spans before, during, and after the COVID-19 pandemic. All interviewers had prior to the study worked as clinical nurses with an advanced degree. They had also previous experience of collecting data through interviews. All the interviewers worked as teachers in the nursing program at the time of the data collection. The interview questions were semi-structured and focused on nursing students´ experiences with age suit simulations, their thoughts regarding ageing and older persons, caring for older persons, related core values, central concepts, and their interactions within the simulation environment. This environment included a highly accessible apartment equipped with WT and other aids. The duration of the group interviews varied, and ranged from 30 to 60 minutes. The group interviews were audio-recorded and transcribed verbatim. In the opening question, the students were asked to describe their experience of simulating in SHC from the viewpoint of their individual persona.

### Analysis

The analysis involved 39 group interviews, amounting to a total of 564 pages. Reflexive thematic analysis (TA) [27, 28] was employed, which is a flexible approach for analysing qualitative data, such as group interviews, to uncover meaningful patterns. The results of this study are described in the form of themes which reveal important aspects of the research topic. Reflexive (TA) aims to go beyond surface description and topic summaries, delving into rich and in-depth storytelling. It encompasses six phases of the analysis, engaging the researcher in an iterative process that involves constant interaction between the data and its interpretation. The analytic table (Table 1) should therefore not be confused with a straightforward approach to the analysis, rather it represents a constant back and forth process. The analysis is described in depth below.

**Table 1:** Table with an overview of the analysis process [27–29]

Phase one - familiarisation	Provided the entry point for analysis and familiarisation with the dataset in an immersive process. It started with data collection and transcribing, reading and listening to the interviews. In addition, rough notes and initial ideas were written down on a separate Word document that served as a reflective journal.
Phase two - coding	Extracts, with both semantic and latent content, relevant to the aim of the study were drawn and labelled with a code. The codes were the building blocks of analysis. The codes were revisited and refined after discussing and reflecting on them with the co-authors.
Phase three - generating initial themes	Through reflective engagement with the data, the codes with shared meaning generated three candidate themes and eight subthemes.
Phase four - review and develop themes	The data extracts and codes in relation to the initial themes were reviewed and the main and subthemes were further developed through a subjective and increasingly interpretative analysis of the data.
Phase five - refining, defining and naming themes	Final phase of naming themes to capture and tell the story, which required an immersive process of refining and defining through back and forth discussions among the authors. A few themes were given new names that more accurately captured the meaning of what was written in the transcripts.
Phase six writing the report	The report was written and finalised.

## Results

**Fig. 1:**
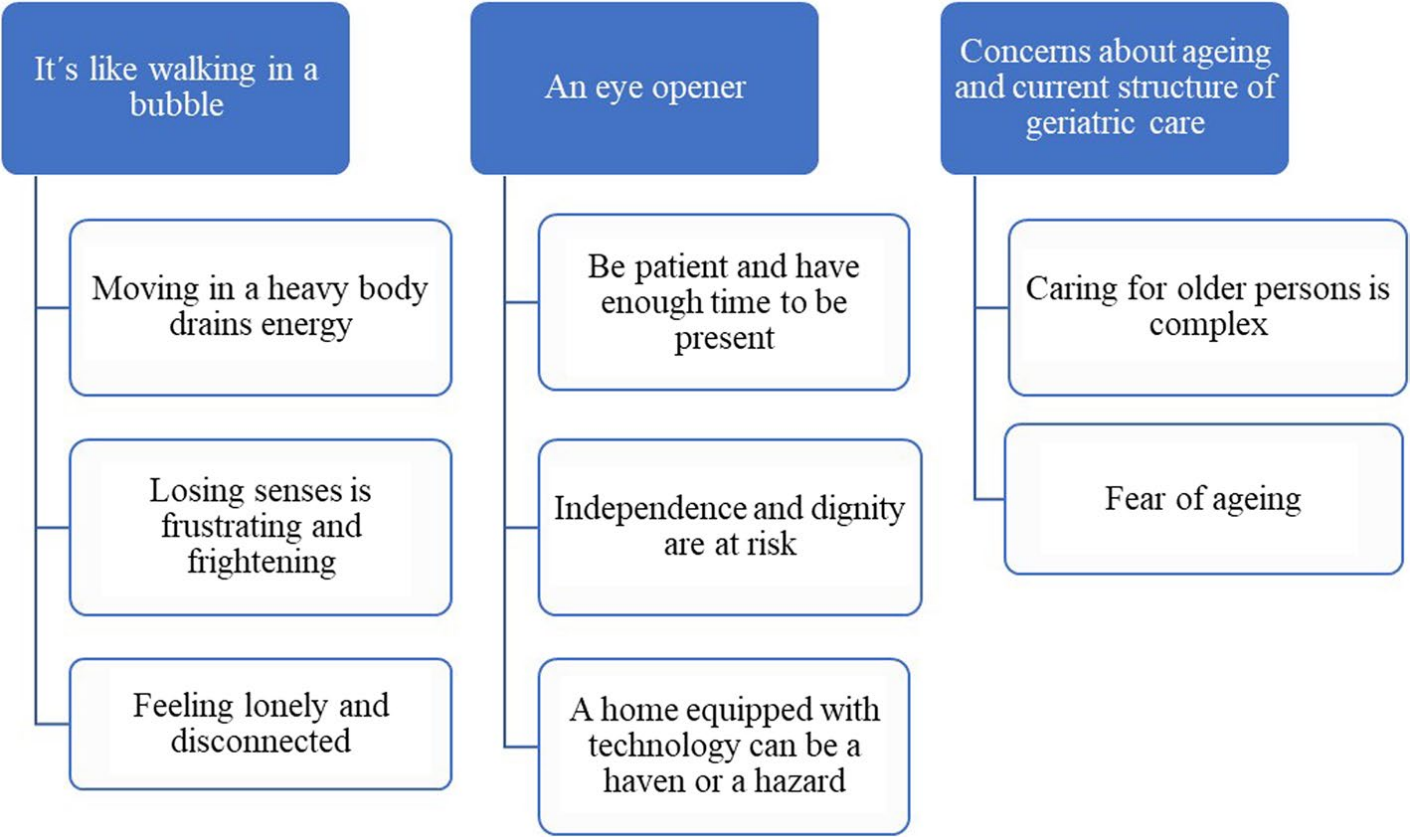
Overview of themes and subthemes

The data analysis of the students´ experiences of partaking in age suit simulation generated three themes and eight subthemes (Fig. 1), which are described in detail below. Representative quotes from the students have been included in the description of each theme and subtheme.

### It´s like walking in a bubble

The theme “It’s like walking in a bubble” includes three subthemes; “Moving in a heavy body drains energy”, “Losing senses is frustrating and frightening”, and “Feeling lonely and disconnected”. This main theme encapsulates various aspects of the simulation related to the students´ experiences of sensory and physical limitations. Adapting to these changes caused feelings of frustration, loneliness and disconnection from others, as said by one student: “*I saw people talking, and I could make out a few words. It was like you were in your own bubble, it was really like you were shut away somewhere*”. The students described a sense of being confined in an unfamiliar body and the challenge of adapting to movements that were painful, slow, sluggish and restrained. Additionally, the loss of sight and hearing intensified the need to concentrate on themselves, limiting their social interaction and engagement. The age suit simulation affected students in ways beyond just wearing a physical suit; it provided an immersive experience, allowing them to identify with older persons´ vulnerability when living with health problems and pain.

#### Moving in a heavy body drains energy

Carrying out ordinary tasks in age suit was experienced as time-consuming and required increased focus and energy, which was in strong contrast to how the students normally felt Finding a comfortable resting position was experienced as difficult, especially for those with knee and back pain. One student expressed “*I just wanted to sit down and rest as often I could; it was pretty heavy and exhausting, it really was. You felt like you were lagging behind because you were so slow and had no energy, just raising an arm was strenuous, it was really tough*”. The struggle to adapt to their disabilities prompted a raised awareness of how ordinary things like picking something up from the floor or using fine motor skills became a real challenge. The students described how they tried and were motivated to find alternative ways to complete the tasks in the home environment while coping with age related impairments and health problems such as stiff joints, heavy legs, insecure gait and pain. Experiencing these energy draining physical limitations affected the students´ mood and they expressed feelings of being vulnerable and exposed: *“Everything goes more slowly, it´s demanding and heavy so obviously you become more vulnerable in a whole other way”*.

An issue often related to feelings of vulnerability and frustration was the realisation that everyday tasks became more difficult or even impossible and took significantly more time than they were used to.

#### Losing senses is frustrating and frightening

Having impaired vision and hearing was described as an emotional experience by the students. Adjusting to impaired vision and hearing loss was described to be frustrating and frightening since it was challenging and accompanied by many trial and error situations. Not being able to see or hear also made students feel vulnerable and dependent, especially since being able to see or hear was something they often took for granted in their own life. The loss of these functions was something they had therefore not reflected on, as expressed by this student: “*I still think this thing with your eyesight feels like the worst impairment you could have. You use your eyes way more than you think*”. Even when compared to pain problems like arthritis or decreased mobility, impaired vision was described as an even stronger limitation in everyday life, causing stress, frustration and exhaustion. The exhaustion was in part associated with a constant search for the best lightning source. Another aspect was that their cognitive decline prompted an increased attentiveness to barriers in the apartment that previously they were unaware of. For example, being able to approximate the distance to a certain item of furniture or finding the correct switch in the kitchen. In one scenario, one student, apart from having health problems, was “blind” and was guided by another student who also experienced cognitive impairments and health problems. The students described being blind as daunting as they had to trust their peers in every step they took. Being blind was experienced to severely reduce the students´ sense of direction, making them more sensitive to barriers in their environment that potentially could hurt them, as expressed by one student: “*Scary being disoriented in an unfamiliar environment. Perhaps you could manage in your own home but not here. It felt like we were walking diagonally when you said we were going straight; it was really unpleasant*”.

Impaired vision and hearing were also related to an experience of stagnation in cognitive abilities. The students expressed that they had to concentrate harder and some even felt like they wanted to “give up”. According to the students, the loss of senses also made it challenging to locate sounds and identify people around them. They experienced that the sudden appearance of a person standing right beside them could startle and throw them completely off guard. The students were in general disturbed by sounds coming from the radio and TV: “*It was just horrible not being able to read, and that damn TV, you couldn’t see it but there was noise everywhere*”. These sounds aggravated the students´ experience of an impaired vision making it even more difficult to focus when they tried to read.

#### Feeling lonely and disconnected

The students mentioned that one consequence of the experience of walking in a bubble was a cognitive decline and feeling lonely even when among others. It was associated with feeling like “zombies” pacing about the apartment disconnected from others. Being lonely and disconnected was often related to the students´ impaired hearing and vision, as these impairments diminished the possibility to communicate. One issue due to the experience of cognitive decline was to even understand if they were being spoken to which made them sometimes feel insecure about initiating a conversation in the first place. Among students who were completely blind, feelings of insecurity and vulnerability were often related to the level of trust in their peers. As one student described it: “*As soon as she left me I thought ‘What’s happening, what are we supposed to do? Where are you going?’* …*and I could not make out what she said and suddenly she was gone. Then I felt insecure*”. The difficulties in communicating sometimes caused students to give up on socialising with their peers, which resulted in them ignoring one another completely. One student voiced this experience as: *“I could barely see, you become lonely. You don´t try and communicate with anyone else you focus on yourself; that was difficult enough*”.

Some students also reported that the experienced cognitive decline made them feel embarrassed to ask for assistance from their peers, which aggravated the social distancing. The disconnection and loneliness evoked feelings of sadness, dependence, loneliness and isolation: “*One thing that I reflected on the whole time wandering around was that there were people everywhere yet I could not see who they were. They passed me by so quickly and I felt alone*”. This highlights a common feeling among the students that when deprived of their senses they felt lonely even though surrounded by their peers. Another aspect of isolation expressed was related to the fear of going outdoors. Staying “at home” was regarded as the safer option because they could not imagine themselves daring to go outdoors. This fear they said added to their feelings of being lonely and disconnected.

### An eye opener

The second theme “An eye opener” and its three subthemes; “Be patient and have enough time to be present”, “Independence and dignity are at risk” and “A home equipped with technology can be a haven or a hazard” reflect aspects of what matters in caring for older persons. Students expressed that the simulations had opened their eyes in a way that increased their ability to better relate to and understand older persons´ experiences. The simulation experience was often more challenging than they had anticipated and opened up the possibility of viewing health care provision from the perspective of the challenges older persons face in their everyday lives, as expressed by one student “*You can actually relate to some extent, it´s not just ‘I understand’. It’s tough, you actually know what it´s like*”. Preserving dignity and independence were seen as important goals and therefore patience and time in caring situations become crucial for inclusive care. Being present was related to the importance of giving the older persons space and time to express themselves.

#### Be patient and have enough time to be present

Wearing the age suit made students realise that ordinary tasks became very time-consuming and caused a multitude of negative feelings such as frustration and a sense of vulnerability. The students´ own experience in the age suit contributed to feeling more respectful of older persons´ everyday struggle. One student described it as: “*You get a deeper understanding and more respect, you understand their situation in a whole new way*”. This deeper understanding was experienced to be useful and implemented in the provision of care in their future profession. They had therefore increased their overall understanding of the importance of giving enough time in regard to the provision of care. An important aspect of giving enough time they described was to be present, not to rush to allow the older person to express themselves and reveal their individual needs. Being present in caring situations was seen by the students as a way of being respectful and more attentive to allow for this time. One student revealed: “*It has affected me a lot today, and it will help me in the future with caring for older persons. I´ll let them take all the time in the world”*. Students felt that the burden of health problems might make older persons lose the initiative to talk about health and well-being as the students themselves had experienced how a cognitive decline affected communication and motivation. A common reflection on observing their peers was that it was difficult to see if the person was suffering or not. With the roles reversed from observing to partaking in the simulations the students described becoming aware of this discrepancy of passive observation and active participation. It was therefore seen as important that they, as future nurses, took their time and listened to what the narratives of older persons could reveal about their everyday lives as those experiences would otherwise go unnoticed.

#### Independence and dignity are at risk

As a result of the self-experienced vulnerability of wearing the age suit the students experienced that dignity and independence now had a new meaning to them. The students related this new understanding of losing abilities to losing a part of one´s identity. The students expressed that they were stunned by the sudden shift from “healthy” to experiencing multiple impairments in the age suit, and they felt concerned about the consequences this loss had on independence. Therefore, the students said, focusing on what matters to the older person is essential to preserve independence. According to the students, one central aspect related to living an independent life was dignity and they raised concerns about the care of older persons having a “one size fits all” approach. The students described the notion of that dignity could be lost if the older person experienced that their resources were not taken into consideration and asserted that it was important for health and social care workers not to “take over”. Therefore, as future nurses, they stated the importance of providing care using a person-centred care (PCC) approach, which was seen as a perspective ensuring the older persons´ needs were accounted for, as described by one student: “*It´s crucial to see the whole individual; not just the disease but how the disease affects this individual person, and fit the care accordingly. ‘You mean person-centred…?’,”Yes exactly”*. The students reflected on the various roles of different social and health care providers and the dissonance that may occur in care provision if they do not share a view of the older person as capable and full of resources despite health problems. The students argued that for PCC to work, an understanding of older persons´ needs on all levels of the health care structure is needed. Students also argued that if care providers were not aligned it could lead to stressful care interactions that could cause the older person to become more passive: “*You take a step back and perhaps don´t want to try because you know the care provider is so stressed out, so you become more passive than you could be*”. However, the students stated that PCC is a team effort. They, therefore, saw age suit simulations as an important opportunity for other caregivers to become more attentive to older persons´ needs, for the sake of preserving dignity and independence. This sentiment was expressed with words to the effect of “*everyone should try this”*. The students experienced that time is precious, and therefore, care providers must be attentive to activities that truly matter to the older person, ensuring their energy is not spent on something that is meaningless. However, the students also argued that it could be a delicate balance between helping too much or too little. The loss of functions, as students felt, was understood to have the potential to affect the older persons’ identity and self-worth. Therefore, students argued for the importance of not only assisting older persons in daily life but also supporting their emotional needs.

#### A home equipped with technology can be a haven or a hazard

The students experienced that the simulation made them increasingly aware of their environment since they identified both positive and negative aspects of using WT. A central aspect related to the environment noted by the students was the importance of proper lighting. This concern was often raised in relation to them describing challenges with impaired vision. A proper light source was experienced as crucial in for example being able to read a piece of paper, communicate with a peer or just navigating safely in the apartment. During simulation, students often felt nervous about falling due to poor lighting conditions and unstable gait. Students experienced that WT have a great potential in assisting older persons to live at home longer because the technology could help them to manage their daily life challenges. They also stressed the importance of adapting the technology to individual needs. If not, the home is at risk of becoming a hazard, which can lead to negative feelings such as frustration and loneliness. For example, if a person with impaired vision cannot distinguish where a certain button to lower kitchen cabinets is located, the whole concept of that technology is useless, which was described by one of the students as: “*It needs to be suited for the individual because if you´re blind you have the need for specific aids whereas if you have arthritis, a hunched back or whatever you need something different*”. Another example in the kitchen was that some students became startled when the cabinets descended from the wall because they could not see or hear it. However other students praised this function as it compensated for the lack of mobility and the pain in their upper limbs and made carrying the kitchen utensils easier. Other aids were also experienced to be of great use to them, for example students with arthritis experienced that it was a relief to get some assistance from the toilet lift when sitting down and standing up from the toilet seat. The students also transferred their understanding of the home environment to hospital health care, discussing the importance of not misplacing items and being more attentive about ensuring proper lighting, and the impact of sounds in the environment. Sounds from the radio or the television were now understood as something that could be potentially very distracting. The students reflected on how often it was assumed by home care providers that older persons like to have the radio or television on, when in fact it could be annoying for those with cognitive impairments. Leaving the radio and television on was also the situation in many service homes, according to their experience. In light of these discussions, the students provided suggestions on how to improve technical solutions as well as other aspects of the environment.

### Concerns regarding ageing and the present structure of geriatric care

The last theme and the two subthemes “Caring for older persons is complex” and “Fear of ageing” conveyed the students´ new insights about the complexity of geriatric care but also their own fear of getting older. The students feared an uncertain future where they would be in a potentially vulnerable position. The students´ insights into complex care needs made them also aware of the older persons´ vulnerability in the health care system: “*You only have 20 minutes, then you clock out, run to the next one; it´s not right. We are working with people, you can´t say that you only have 20 minutes and therefore you don´t get to shower properly because someone else is waiting”*. These experiences illustrate what the students would refer to as older persons being caught in a system they have little or no influence over.

#### Caring for older persons is complex

The students reported that experiencing health problems first-hand through the simulations gave them a deepened understanding of older persons' complex care needs. One student expressed her experience in these words: “*It is so difficult working with multiple care needs, like you have this feeling something is wrong but they [the older person] doesn’t tell you*...*and now I felt pain in my knee but I also had trouble hearing, it’s like everything is wrong but I can’t find the right words to explain what the real problem is. It´s so hard to care for such a complex person”*.

They also related this understanding to a broader perspective describing how the nurse was just one of many professions and providers that the older persons are dependent on in the health and social care system. Furthermore, the context of providing care in someone´s home requires good communication skills but also a positive attitude towards older persons. From their own experience of having cognitive impairments, they stressed the importance of continuity in building trusting relationships. They recognized that living with health problems makes older persons more vulnerable, and therefore, allowing a care provider you don´t know into your home can be terrifying. The students described a frustration where there is an inherent imbalance regarding decision-making and resources between health and social care providers and older persons. The age suit had made the students aware of the value of time, and the students often reiterated that this extra time was something they rarely saw in clinical practice. The students argued that a serious problem in the health care system were limited resources. This problem was often exemplified as a tight time schedule and a lack of adequate training among care providers. They found this troublesome since quality care requires a team effort, and with limited resources time should be spent on what matters to each individual. However, the simulations made them aware that being sympathetic and genuine can make a huge difference in building a relationship, and it requires no extra time. Some students stated that they had observed care providers act disrespectful towards older persons during their clinical practice and these observations contributed to their negative view on geriatric care. The students now felt more worried for the future of geriatric care because they feared care providers did not have the proper training, attitude or skills to respond to complex health care needs. One student conveyed this in the following quote: “*It feels like health care provision is not taken as seriously as back in the day, that you don´t feel the same responsibility and respect when caring for older persons, and that worries me actually*”.

Even though the students were worried about the future they expressed hope that WT could be of great help to compensate for a tight time schedule and the complexity of care. But at the same time, students were concerned about issues related to this technology such as integrity, especially when using cameras, and user-friendliness. They concluded that in implementing WT, individual needs such as poor vision and digital literacy must be taken into consideration:

*“I am not so technically skilled and with all the impairments you had it became even more difficult to figure out what you were supposed to do with it* [WT]*. And it was very difficult to make out the sounds from the iPad and it was hard to read.”*

The students also raised concerns about older persons tripping over cables or accidentally disconnecting sensors. There was also the concern about technology increasingly replacing actual human contact. Implementing WT was described as a balance between the older persons´ and care providers´ needs. The students stressed that adapting WT to individual needs requires a unified understanding of complex care needs from all health care providers. The students conveyed another concern which was the lack of continuity in health care provision, and they said that those in charge, such as politicians and care managers, did not have knowledge about older persons´ needs. Nurse assistants were often mentioned as the care givers closest to the patient and therefore were thought of as an important group that could benefit from age suit simulations. The students argued that since the age suit simulation had been an eye-opener for them, the same could be true for anyone in the health care system in reaching a deeper understanding of older persons' complex care needs.

#### Fear of ageing

Students frequently raised concerns about the future of geriatric care in relation to their own ageing process. The fear of ageing was, in part related to the pain of physical limitations and, in part, related to social aspects such as becoming lonely, dependent, and isolated. The students thought society at large did not value geriatric care enough and they worried that this would impact what resources would be available for themselves in the future. As future older persons they could think of few positive aspects of reaching old age, even though they hoped technology and improved aids would facilitate healthy ageing, independence, and remaining at home. However, many still feared an uncertain future and the disabilities they would have to struggle with. This feeling was described by one student: “*Fear of becoming limited and not being able to get through the day, not knowing how…well fear of not knowing what disabilities you will have, you don´t know what the future holds*”. The student felt that uncertainty was among the worst parts of growing or becoming older as the age suit had given them an indication of what they might expect as older persons. The negative feelings about ageing were also said to be reinforced by encounters with older persons who described ageing as something terrible or unworthy. Those who were already worried about ageing experienced that the simulations added to those emotions and they described fear of trusting care givers to support and care for their needs as older persons. The students experienced however that their negative feelings prompted a greater understanding of why older persons sometimes express feelings of distress or wanting to end their life. The students also reasoned that in the age simulation they transitioned quickly from being young and capable to experience multiple health problems. They noted that in real life this transition takes more time and therefore there is a chance of getting used to the decline in capabilities and health. The simulations also prompted students to converse about the importance of staying healthy during the ageing process.

## Discussion

The main results of this study show that nursing students find age suit simulation valuable for increasing their understanding of older persons´ care needs through the raised awareness of functional challenges in everyday life and because it enhances their perspectives and understanding regarding ageing and associated health problems. Another study [30] conclude that age suit simulation is an effective educational tool that increased health care professionals´ awareness of their feelings and knowledge about age-related limitations. Eppich and Reedy [31] argue that research on simulations should focus on how they work rather than whether they work. They also stress the importance of qualitative methods being based on learning theory.

It was obvious that the students found the simulations challenging in many ways, and they were often more demanding than expected. The physical challenges of age suit simulation have previously been highlighted [32] as well as how the simulation affects students´ emotional state and increases empathy towards older persons [21]. The physical challenges as well as the students´ emotional response to the simulations previously mentioned resonate with the findings of students being both frustrated and humbled by the simulation. Even though the students were not specifically asked about empathy, the results indicate that students felt more sympathetic towards older persons´ health problems.

A possible explanation of why the age suit simulation evokes strong emotions is that it provides a unique and immersive experience of living with health problems from the patient´s point of view and this is unlike anything else the students have experienced during their training. Demirtas and Basak [33] study shows something similar in regards to students becoming emotional during the simulations. The immersive experience of losing function and autonomy can be described as a direct confrontation with perceived ideas of ageing, older persons and age-related health problems. A review article on Experiential Learning Theory (ELT) describes how emotions influence students´ learning process in that students´ emotional state dictates what information is retained in their memory. In short, attention is directed at what resonates with one´s emotions at the time [34].

Simulating with the age suit at SHC, we argue, resonates with the concept of a *concrete experience* in ELT, which Kolb and Kolb [35] describe as an experience out of the ordinary that challenges previous convictions and ideas. In a review article, ELT is described as a hands-on experience where learners are active and engaged socially, intellectually and physically, which explains the embodied nature of ELT [36]. The concrete experience is therefore an essential part of experienced-based learning [35]. Emotions are often neglected in simulation training and the focus is on logic and problem-solving. However, emotions play an important role in learning. For example, negative emotions can both be a barrier as well as enhance learning and motivation [37]. This relates to our results describing students´ fear of ageing. Arguably, if a person, prior to the simulations, feels anxious or scared of age-related problems, it is possible in our opinion that those emotions will be enforced by experiencing health problems in age suit simulation. In the present study, the students´ feelings were often an important starting point for insightful reflections that were transferred into other perspectives. Based on the results of the present study, it can be argued that implementing age suit simulation must be done at the “right” time in the curriculum for students to benefit from them. This is because nursing students need a theoretical and clinical reference frame to relate to. Kolb and Kolb [35] argue that in order to make sense of the world and our experiences, both concrete experience and abstract thinking are needed. For a dialogue to occur there is a need to reflect on one´s actions. Students are more aware of how vulnerable the patient is, which is important for health care provision and the practice of PCC.

Another important finding, we argue, is the students´ intention of including their new knowledge about functional challenges in future interactions with older persons in a clinical setting. This is similar to a study by [38] that concluded that age suit simulation is effective in raising students´ awareness of functional challenges among older persons as the age suit replicates normative values for older persons based on mobility and balance metrics. Even though metric testing was not part of this study, the nursing students often described their experience of being able to relate to older persons´ challenges as it was an embodied experience. Interestingly, our findings not only highlight functional awareness among the students but also attentiveness to older persons´ feelings. Another study [38] also showed students experienced guilt and sadness because they realised they had not had the proper knowledge or demonstrated attentiveness to older persons´ needs, which corresponds with our results of students raising the complexity of geriatric care. Illuminating the complexity of geriatric care resonates with previous studies calling for improved geriatric education [17, 19].

The study also emphasises a critical perspective on WT, and students find it to be both a helpful and important feature of the provision of modern health and social care as well as a potential threat to safety and integrity. Interestingly a recent article exploring attitudes towards the use of technology over different generations (from 30-79 years old) showed similar results. The raised awareness of technology use in health and social care situations in the present study therefore aligns with the perspectives of potential end users, which arguably is an important aspect of implementing and understanding WT [39]. Technological literacy has also been highlighted as an essential skill for nurses. A scoping review on this topic revealed that nursing students´ technological literacy in general is heterogeneous. The authors of the review stress that because nurses are the persons closest to the patient, they are the ones most aware of the patients´ daily needs and therefore have a significant role in developing and implementing technology. Research and pedagogical development regarding technical literacy are essential for both educators and nursing students [6]. These findings resonate with the accessible and technically advanced simulation environment at SHC, signifying the importance of students interacting with WT and other technical aids to improve their technical literacy but also to develop critical thinking regarding its use and relevance for the individual.

Beyond expectation was how emotional the students were regarding different eye impairments. Vision is the most dominant human sense and most people of old age will experience some kind of eye condition. The students described communication as a means of being involved and active. Not being able to communicate is regarded as posing a risk of being separate from a social context. Impaired vision therefore has several implications. According to the WHO´s World Report on Vision [40], societies are built on the ability of sight; thus integration, everyday activities and social interactions are dependent on functioning vision. The current study touches upon an important aspect of home care provision to older persons, namely informal caregivers. This aspect is experienced in the scenario where one student is blind and is assisted by a peer who also suffers from health problems. According to a study on informal caregivers, 15 % of the adult population in Sweden provide care as informal caregivers, which is significant given that Sweden is regarded as a country with a liberal welfare system [41]. In light of the students´ experience of sensory impairment these specific impairments, blindness in particular, will be the focus of an upcoming study from the authors.

In line with the results in the current study, another study [42] showed that students experienced frustration, anger, loss of independence, fear and vulnerability. They also reported, in correlation with our study, that students with work experience expressed increased understanding of the emotional response from older persons as a result of partaking in the simulation. Increased understanding can be seen as an underlying focal point throughout the themes. In our study, students described their intention to put their new knowledge to use in clinical practice. This intention is often related to improving the quality of care but also to preserving older persons´ dignity. Another study [43] suggest nursing students mostly agree that dignity is a skill that can be taught in education through *experience*. Based on the findings in the current study it can be argued that simulation is a relevant complement to didactic education. It is about balance, as Kolb and Kolb [35] explain that for learning to occur there must be a balance between activity and reflection, where both theory and experience are important pieces of a puzzle. Future studies may include age suit simulation used by other health care professionals and settings.

### Strengths and weaknesses

Braun and Clarke [29] stated that the aim of reflexive TA is to make sense of patterns of meaning across a dataset. Quality in reflexive TA is reflected in complex and multifaceted themes, and as Byrne [44] points out, quality in reflexive TA is not represented by providing a single right answer. A strength of the present study was the collaboration between the researchers in exploring and achieving rich interpretations of the material. Regular peer meetings to discuss thoughts and ideas on the evolving data to strengthen a study´s trustworthiness which are in line with a previous study [45] on quality in TA. On the issue of quality, Braun and Clarke [46] state that if themes could be created beforehand or merely reflect a research question, it is a sign of poor-quality reflexive TA. They refer to these themes as topic summaries. Rather, themes should be generated which could bring together seemingly unrelated topics [46]. In the present study, two of the authors were familiar with reflexive TA and could provide valuable insights, which was helpful in generating the themes. The nursing students were not invited to reflect on the analysis, which could be seen as a limitation as there may be gaps of understanding. Another possible limitation is author bias during data collection as the interviewers were working as teachers in the nursing program at the time of the study and therefore had a relation to the students. However, the interviewers reminded the students several time that participation was voluntary and that they could withdraw their participation at any time. Data was collected separately by three of the authors using the same interview guide. There is still little research about objective quality measures in thematic analysis [45]. Therefore, trustworthiness measures as described by Graneheim and Lundman [47], such as credibility, dependability and transferability, were also used. Interviewing all students and not excluding anyone from participation is considered to add to the study´s credibility as is the use of multiple perspectives on the investigated phenomenon. Credibility was also enhanced by using representative quotes from the students. Dependability relates to the stability of the data collection, especially if it is performed over longer periods of time [47] In the present study, no significant alterations were made to the research design or questions, meaning that students were asked the same question at the same point in time of their education. Transferability relates to the extent to which the results can be generalised to other contexts and is facilitated by providing a clear and transparent description of the study design and considerations as well as by presenting the results in a logical and precise manner [47]. However, ultimately it is up to the reader to judge to what extent the results can be transferred to different contexts. NVivo version 1.7 was used to facilitate the process of coding due to the large data and the programme helped the researchers in organising and sorting data but was not used to perform or guide the analysis. The benefits of using NVivo for large amounts of data in thematic analysis is confirmed in a previous study [45]. Replicating age-related health problems in age suit simulation among young adults also has limitations. In another study [38] the age suit was considered to replicate the age-related risk of falling reasonably well in both male and female students. However, among some of the male students, the age suit had little or no effect on their physical capabilities, which was related to men in general having larger muscles. Therefore, it is possible that there are gender differences in age suit simulation related to physical capabilities, which may have influenced the present study.

## Conclusions

This study concludes that taking the point of view of “an older person” in age suit simulation became an embodied and eye-opening experience for nursing students. The results highlight that cognitive loss was especially difficult for the nursing students to handle. In light of the increased shift from care provision in institutions to home care, the context for the simulation and the simulation intervention, affect nursing students´ attentiveness to older persons´ needs resonates with PCC, which is a crucial concept of home care provision. After the simulations, the students felt more aware of older persons´ vulnerable position in the health care system and stressed the importance of PCC as a measure to safeguard older persons´ dignity and independence. The study design also illustrates the pedagogical value of including ELT in age suit simulations because it requires students to critically reflect on their preconceived ideas of aging, older persons, health problems and providing care to older persons. This study therefore illustrates how age suit simulation can be an important part of nurse education in preparing students for the complexity of geriatric care, but could also be of relevance to other health care professionals .

### Supplementary Information

**Additional file 1: Appendix 1.** Interview guide.

## Data Availability

The datasets used and/or analysed during the current study are available from the corresponding author on reasonable request.

## Abbreviations

WT: Welfare Technology
GERT: GERonTologic age suit
SHC: Skaraborg Health Technology Center
PCC: Person-Centred Care
ELT: Experiential Learning Theory
