# Supplementary material for: ”It´s like walking in a bubble”, nursing students´ perspectives on age suit simulation in a home environment – group interviews from reflection seminars
Source: BMC Nurs. 2024 Feb 16;23:124. doi: 10.1186/s12912-024-01792-5 (PMC10870535; doi:10.1186/s12912-024-01792-5)
Supplement: Supplementary file 1 — Additional file 1: Appendix 1. Interview guide. [file 12912_2024_1792_MOESM1_ESM.docx]

# Interview guide

**Introduction** (roundtable reflection – each student provides their reflection)

* Describe your experience of simulating with an age suit in SHC
 (how does it correspond to the participant´s expectations, conceptions and **apprehensions?)**

**Questions (open group discussion)**

*** Describe your thoughts and feelings regarding being a guide or provide guidance**

*** Describe your thoughts and feelings regarding aging, older persons and caring for older persons**

*** In what way has the age suit simulation affected your view on:**

**- Aging and living with health problems**

**- Older persons**

**- Providing care to older persons**

**- Significance of the simulation environment (highly accessible apartment with welfare technology)**

**- Future career orientation in the profession**

*** Describe your thoughts and feelings when observing the simulations**

*** How do you relate your experience of simulation to central concepts of caring in regard to
 aging, older persons and caring for older persons?**

*** How do you relate your experience in the age suit simulation to the nurse´s core
 competencies in regard to aging, older persons and caring for older persons?**

**Closure** (roundtable reflection – each student provides their reflection)

* What is your take away from this age suit simulation and describe how it has influenced
 your view on aging, older persons and future care of older persons?
